# Dual Functional Ultrafiltration Membranes with Enzymatic Digestion and Thermo-Responsivity for Protein Self-Cleaning

**DOI:** 10.3390/membranes8030085

**Published:** 2018-09-19

**Authors:** Anbharasi Vanangamudi, Ludovic F. Dumée, Mikel C. Duke, Xing Yang

**Affiliations:** 1 Institute for Sustainable Industries and Liveable Cities, College of Engineering and Science, Victoria University, P.O. Box 14428, Melbourne, VIC 8001, Australia; mikel.duke@vu.edu.au; 2 Institute for Frontier Materials, Deakin University, Waurn Ponds, Geelong, VIC 3216, Australia; ludovic.dumee@deakin.edu.au

**Keywords:** thermo-responsive, ultrafiltration, enzymes, self-cleaning, nanofibers

## Abstract

Controlling surface–protein interaction during wastewater treatment is the key motivation for developing functionally modified membranes. A new biocatalytic thermo-responsive poly vinylidene fluoride (PVDF)/nylon-6,6/poly(*N*-isopropylacrylamide)(PNIPAAm) ultrafiltration membrane was fabricated to achieve dual functionality of protein-digestion and thermo-responsive self-cleaning. The PVDF/nylon-6,6/PNIPAAm composite membranes were constructed by integrating a hydrophobic PVDF cast layer and hydrophilic nylon-6,6/PNIPAAm nanofiber layer on to which trypsin was covalently immobilized. The enzyme immobilization density on the membrane surface decreased with increasing PNIPAAm concentration, due to the decreased number of amine functional sites. An ultrafiltration study was performed using the synthetic model solution containing BSA/NaCl/CaCl_2,_ where the PNIPAAm containing biocatalytic membranes demonstrated a combined effect of enzymatic and thermo-switchable self-cleaning. The membrane without PNIPAAm revealed superior fouling resistance and self-cleaning with an R_PD_ of 22%, compared to membranes with 2 and 4 wt % PNIPAAm with 26% and 33% R_PD_, respectively, after an intermediate temperature cleaning at 50 °C, indicating that higher enzyme density offers more efficient self-cleaning than the combined effect of enzyme and PNIPAAm at low concentration. The conformational volume phase transition of PNIPAAm did not affect the stability of immobilized trypsin on membrane surfaces. Such novel surface engineering design offer a promising route to mitigate surface–protein contamination in wastewater applications.

## 1. Introduction

Non-specific surface–protein interactions at the membrane interface during ultrafiltration (UF) leads to permanent fouling, by accumulation of protein contaminants on the surface or pores of the membrane [1]. Membrane fouling by proteins block the membrane pores and eventually form cake layer that rapidly decline membrane permeability, increase the cleaning frequency and reduce membrane performance [2,3]. One of the most adaptable methods to decrease fouling and self-clean the membranes is to modify the membrane surface functionalities by incorporating self-cleaning materials such as hydrophilic copolymers [4,5], amphiphilic copolymers [6], zwitterionic compounds [7], metal oxides [8], biocatalytic enzymes [1,9], and responsive materials [5,10,11]. Self-cleaning materials are a class of materials with intrinsic ability to remove any contaminant from their surfaces via various mechanisms [12].

Enzymes are biocatalizers that act as biochemical catalysts of specific substrates to produce individual products. Proteolytic enzymes have attracted attention as self-cleaning compounds that can breakdown and remove the protein foulants from the membrane surface [1,13]. To overcome self-hydrolysis of free enzymes in solution leading to instability, deprived performance and poor reusability [14], enzymes may be immobilized onto suitable substrates. The nature and properties of the substrates play an important role in enhancing the loading of enzymes, enzyme stability and its activity over time and cleaning cycles [15].

Electrospun nanofibers, owing to their high surface-to-volume ratio, are considered to be one of the most appropriate substrates for enzyme immobilization providing high loading of enzymes and improved stability [16], as well as great structural versatility and facile control on surface chemistry [17,18]. The nanofiber membranes possess high porosity and pore interconnectivity that provide low hindrance to mass transfer making it suitable for filtration [19,20]. The activity of enzyme immobilized onto nanofibers was found to be greater than that of the activity of enzymes immobilized onto commercially cast membranes, owing to the high surface area providing more active sites for enzyme immobilization [9,21,22]. Furthermore, the enzyme immobilized onto nanofibers demonstrated good operational reusability. For example, trypsin immobilized onto polyethylene terephthalate (PET)/poly (lactic acid) (PLA) nanofiber mats and chitosan nanofibers presented 80% (eleven cycles) and 97% (five cycles) reusability, respectively [23,24]. Nanofibers are typically used as the top functional layer together with a support layer underneath, during the treatment of complex wastewater [25]. Despite showing enhanced membrane antifouling performance and enzyme reusability, the reported biocatalytic UF membranes exhibited low permeability [1,26,27]. Thus, biocatalytic fouling resistant membranes with stable enzyme immobilization onto the surface and altered pore structure offering high permeability and long-term operational stability are desired. Since enzymes are susceptible to loss in activity over time [9,28], an additional self-cleaning material that provide facile membrane cleaning may be incorporated to achieve enhanced performance.

**Figure 1:**
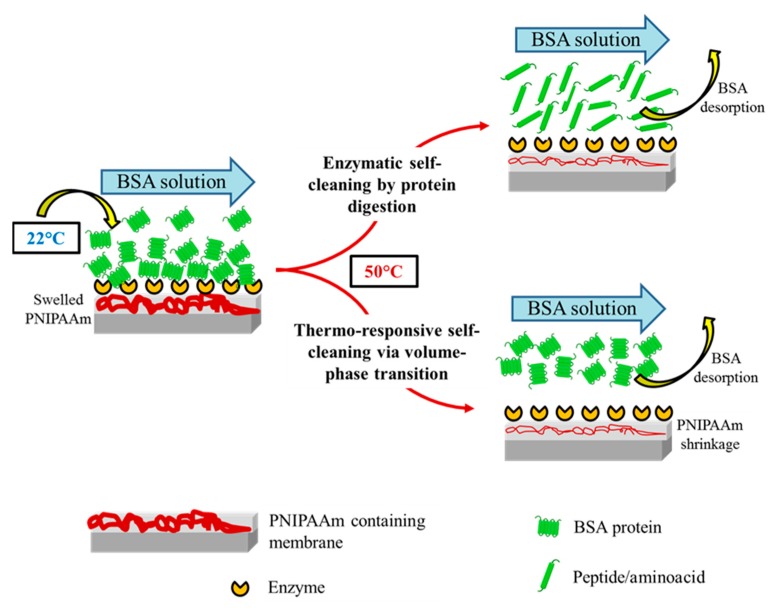
Conceptual schematic of self-cleaning biocatalytic and thermo-switchable membrane.

Thermo-responsive polymers are considered among the promising antifouling materials that offer facile temperature-based cleaning for membranes [29]. With a lower critical solution temperature (LCST) of about 32 °C in an aqueous solution, poly(*N*-isopropylacrylamide) (PNIPAAm) is a well-recognised temperature-sensitive polymer [30,31]. Below LCST, the PNIPAAm polymer chains are more hydrophilic having an extended conformation in water and above LCST, they become less hydrophilic forming a dehydrated compact structure exhibiting a sharp reversible volume-phase conformational transition providing strong inherent washing force. On one hand, the self-cleaning behaviour of the PNIPAAm containing membrane could be attributed to the enhanced hydrophilicity below its LCST, thus facilitating foulants desorption from the surface. For example, PNIPAAm grafted polydopamine/PET UF membranes recovered 90% of the initial flux at 20 °C compared to unmodified PET membrane that showed only 76% flux recovery, ascribed to the enhanced surface hydrophilicity [29]. Similarly, a flux recovery of 92% was achieved for the poly (vinylidene fluoride) (PVDF)/TiO_2_-g-PNIPAAm nanocomposite membranes compared to 47% flux recovery for the control PVDF membranes at 23 °C [32]. On the other hand, the thermo-switchable characteristic of PNIPAAm providing strong inherent washing force was exploited to remove the membrane foulants in UF, exhibiting self-cleaning property. For example, the polyethylene membrane onto which PNIPAAm was grafted, showed 97% flux recovery via applying a temperature-change (25 °C/35 °C) cleaning method to the bovine serum albumin (BSA) fouled membranes [33]. Similarly, the PNIPAAm-grafted ZrO_2_ membrane showed 80% flux recovery after temperature-change cleaning (25 °C/35 °C) of BSA fouled membranes [34]. However, the combined self-cleaning effect of PNIPAAm and biocatalytic enzymes has not been explored so far and the impact of one material on the other with respect to filtration and self-cleaning effect was not investigated. In this study, a new biocatalytic PVDF/nylon-6,6/PNIPAAm composite UF membrane was fabricated by covalently immobilizing trypsin (TR) enzyme onto functional nanofibrous surface of PVDF/nylon-6,6/PNIPAAm membrane, to achieve dual functionality of protein-digestion and thermo-responsivity for self-cleaning effect. The structural and functional properties of the as-prepared composite membranes were investigated and correlated to the membrane performance in UF fouling experiments with intermediate temperature cleaning. Also, the impact of thermo-switchable volume-phase transition on the stability of immobilized enzymes was studied. Figure 1 shows the schematic of membrane self-cleaning using enzymes and thermo-responsive PNIPAAm polymer via protein-digestion and volume phase transition mechanisms, respectively. 

## 2. Experimental Section

### 2.1. Materials 

PVDF Kynar 761 with a melting point 165–172 °C was purchased from Arkema Pte. Ltd. (Singapore). Trypsin (from porcine pancreas) was purchased from Wako pure chemical industries Ltd. (Osaka, Japan). PNIPAAm, (Mw 113 g/mol), polyamide-6,6 (nylon-6,6) (Mw 262.35 g/mol), poly(vinylpyrrolidone) (PVP-K-40) (Mw 40,000), 1-ethyl-3-(3-dimethylaminopropyl) carbodiimide (EDC), BSA (Mw 66 kDa) as model protein, *N*-hydroxysuccinimide (NHS), formic acid (>95%), *N*,*N*′-dimethylacetamide (DMAC) (99.8%), trichloroacetic acid (TCA) (99%), ethanol (75%), sodium chloride (NaCl), glycerol (>99.5%) and calcium chloride (CaCl_2_) were purchased from Sigma Aldrich (St. Louis, MO, USA) and was used as received. Deionized (DI) water was obtained from the Milli-Q plus system (Millipore, Bedford, MA, USA) and used in all experiments. 

### 2.2. Preparation of PVDF/nylon-6,6/PNIPAAm Membrane

The thermo-responsive PVDF/nylon-6,6/PNIPAAm composite membrane was prepared using a similar method used in our earlier study [5]. Concisely, the preparation of composite membrane was carried out using three consecutive steps, (a) construction of thermo-responsive functional nanofiber mat by electrospinning a mixed solution of two different PNIPAAm concentrations (2 and 4 wt % PNIPAAm) and 10 wt % nylon-6,6 in formic acid, at 0.25 mL/h flow rate and 17 kV voltage with 150 mm tip to collector distance, (b) conventional casting of the PVDF dope solution prepared by continuous stirring of 8 wt % PVP and 18 wt % PVDF in DMAC solvent at 50 °C overnight, on to the nanofiber mat and (c) immersion of the cast and nanofiber layers together into a coagulation tank of DI water to remove the residual solvent via phase inversion. Further, the post-treatment of nascent membranes was performed by immersing them in to a mixture of ethanol, glycerol and DI water in the ratio 1:2:2 (vol %) and was dried finally before characterisation. Also, the control PVDF/nylon-6,6 membrane was fabricated without the addition of PNIPAAm.

### 2.3. Preparation of Biocatalytic PVDF/nylon-6,6/PNIPAAm Membranes

The TR enzyme immobilization on to the as-prepared membranes with no PNIPAAm (PN0), 2 wt % (PN2) and 4 wt % (PN4) PNIPAAm were attained by EDC/NHS immobilization reaction using a similar method used in our previous study [9], to form PN0-TR, PN2-TR and PN4-TR membranes, respectively. Firstly, 1 mg/mL TR solution was reacted with EDC/NHS (4:1) aqueous solution for about 1 h at room temperature, to activate the enzyme carboxyl groups. Secondly, the activated enzymes were covalently attached onto the PN0-TR, PN2-TR and PN4-TR membranes by reacting with the primary amines on the membrane surface for 12 h at 4 °C. Finally, the absorbed TR was removed by rinsing the membranes with DI water. The decrease in enzyme concentration in solution before and after contact with the membrane was used to calculate the enzyme immobilization efficiency.

### 2.4. Membrane Characterization

Scanning electron microscopy (SEM) (SUPRA 55VP, ZEISS, Jena, Germany) was used to study the surface morphology of the as-prepared biocatalytic membranes. The accelerating voltage was set to 5 kV with 10 mm working distance for the observation. The membrane samples were prepared prior to observation by sputter coating them using Leica EM ACE600 (Leica microsystems, Sydney, NSW, Australia), in high vacuum with a gold layer of 5 nm thickness. The observed SEM images were used to evaluate the average nanofiber diameters of the membranes using ImageJ software. Porometer 3Gzh (Quantachrome, Boynton beach, FL, USA) was used to measure the membrane pore size and its distribution. The membranes (25 mm diameter each) were first wetted with Porofil™ liquid and positioned in the sample holder after which it was exposed to 6.4 to 34 bar pressures for wet and dry run. The measurement was carried out three times for each membrane to obtain the average pore size. CAM101 optical contact angle meter (KSV Instruments, Helsinki, Finland) was used to measure the dynamic water contact angles (CA_w_) of the as-prepared membranes and to investigate the switchable surface hydrophilicity at 22 °C (below LCST) and 50 °C (above LCST). The required temperature of the membrane samples was achieved by adjusting the voltage of the source meter connected to the heating pad on which the samples are mounted. Prior optimisation of corresponding temperatures and feed voltages of the heating mats were established before mounting the heating pad on the contact angle meter. The measurement was performed by pasting rectangular strips of each membrane sample on to the sample stage and dispensing 4 µL water droplet onto the membrane surface through a needle. Each measurement was recorded every 5 s over the duration of 60 s.

### 2.5. Quantification of Immobilized TR and Its Activity against BSA

UV–Visible spectrophotometer (Model UV-1800, Shimadzu, Columbia, SC, USA) was used to measure the decrease in TR concentration of the test solution before and after filtration experiments at the wavelength of 280 nm, owing to its simplicity, reliability and convenience. The immobilized TR surface density of the thermo-responsive composite membranes was calculated similarly to the method reported in literature [1]. Furthermore, the enzymatic activities of biocatalytic thermo-responsive membranes and free TR were calculated by measuring their hydrolytic activities via previously described method using 1 wt % BSA solution as the substrate [9]. Briefly, the immobilized and free TR were first reacted with the BSA solution for up to 1 h at 37 °C after which the reaction was terminated using 5 wt % TCA and then centrifuged at 2000× g to measure the absorbance of the supernatant containing hydrolytic products using UV–Visible spectrophotometer (280 nm). The supernatant of the centrifuged solution after similar reaction without TR was used as the blank. In this study, 0.1 increase in absorbance of the hydrolytic products represents one digestion unit (DU) that denotes an increase in the amount of substrate digested by the enzymes via hydrolysis. However, the measured hydrolytic activity of the immobilized enzymes was normalized to 100% as a benchmark, based on the literature that showed superior activity and operational stability of the enzymes immobilized on to nanofibrous substrate [16].

### 2.6. Fouling Studies

A cross flow UF system (42 × 10^−4^ m^2^ effective area; 12.6 cm/s flow velocity) was used to evaluate the antifouling and self-cleaning properties of the biocatalytic thermo-responsive membranes. To simulate a practical fouling environment in wastewater treatment, a complex synthetic feed solution containing 1 mg/mL BSA (model protein), 1 mM CaCl_2_ and 7 mM NaCl in DI water with pH 7.8 (optimum TR pH range 7.5–8.5) was used in this study [35]. The addition of NaCl and CaCl_2_ to the protein feed solution greatly increased the potential for surface fouling and simulated a practical fouling environment. It was demonstrated that a thicker and more compact fouling layer was formed on the membrane surface through the calcium-induced protein aggregation via (a) forming protein−Ca^2+^−protein complexes and (2) intramolecular electrostatic shielding of the protein negative charges by Ca^2+^ [36]. Although the current study focused on protein rich synthetic solution, future work studying the novel membrane’s ability to perform in such complex real water matrices for specific applications could be performed. Initially, each membrane was compacted at 120 kPa for 10 min at RT using DI water and then exposed to DI water containing 7 mM NaCl at 100 kPa for 15 min to measure the clean water permeance (*P_w_*) in L m^−2^ h^−1^ calculated by the following equation:

(1) $$P_{w}=V/\left(A\times t\times p\right)$$

where *V* stands for the permeate volume in L, *A* stands for the membrane area in m^2^, *t* stands for the permeation time in h and *p* stands for the constant pressure (1 bar). Each cycle of the 2 cycle UF experiment includes the filtration of the as-prepared feed solution at 22 °C for 1 h followed by an intermediate temperature cleaning with DI water at 22 °C for 15 min. The cycle number was denoted by ‘n’. The fouling studies were carried out by performing the UF experiment three times for each of the membranes and was averaged to ensure reproducibility. The rate of permeance decline (R_PD_) after each cycle was determined as a measure of protein fouling using the equation,

(2) $$R_{PD}\;\left(\%\right)=\left[1-\left(\frac{P_{e\left(n\right)}}{P_{w}}\right)\right]\times 100$$

where *P_e(n)_* stands for the final feed permeance in *n^th^* cycle. Further, the membrane self-cleaning property was studied by calculating the permeance recovery after the intermediate temperature cleaning at 22 °C, using the equation,

(3) $$PRR\;\left(\%\right)=\frac{P_{w\left(n\right)}}{Pw}\times 100$$

where *P_w(n)_* stands for the clean water permeance in *n^th^* cycle. Also, the fouling parameters namely irreversible fouling (*IF*), reversible fouling (*RF*) and total fouling (*TF*) for each cycle was computed by the following equations:

(4) $$IF=\left[P_{w\left(n-1\right)}-P_{w\left(n\right)}\right]/P$$

(5) $$RF=\left[P_{s\left(n\right)}-P_{e\left(n\right)}\right]/P$$

(6) TF=IF+RF

where *P_s_* stands for the initial feed permeance in each cycle and *P_e_* stands for the final feed permeance in each cycle. Finally, the membrane surfaces were visualised after 2 cycles of filtration using SEM and the antifouling and self-cleaning properties of the enzyme immobilized membranes with and without PNIPAAm was compared. Further, to investigate the combined antifouling and self-cleaning effects of protein-digestive enzymes and thermo-responsive PNIPAAm, 2 filtration cycles each including 1 h filtration of the as-prepared feed solution at 22 °C followed by an intermediate temperature cleaning with DI water at 50 °C for 15 min were also performed and their respective R_PD_ was calculated for comparison.

### 2.7. Storage Studies and Effect of Thermo-Responsivity on Enzyme Stability

The storage study for the biocatalytic membranes were conducted by storing them under refrigeration at 4 °C and RT (22 °C) up to two weeks during which the enzyme activity was measured at regular intervals. Further, the effect of thermo-switchable volume phase transition of the PNIPAAm on enzyme stability was examined by measuring the hydrolytic activities of the as-prepared membranes (a) before and after treating the membranes for 5 min at 50 °C and (b) over six consecutive reuse cycles before treating the membranes for 5 min at 50 °C and after the treatment. These studies were conducted to investigate if the volume phase transition during thermo-switchable cleaning affects the stability of enzymes immobilized on to the membrane surfaces; 5 min treatment at 50 °C is exposing the membrane samples to DI water maintained at 50 °C and mild stirring at 100 rpm for 5 min.

## 3. Results and Discussion

### 3.1. Enzyme Distribution on Membrane Surface

**Figure 2:**
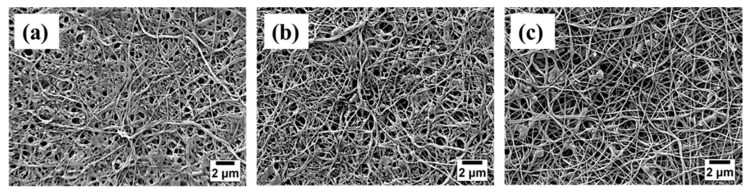
SEM images of biocatalytic membranes with (**a**) no PNIPAAm (PN0-TR); (**b**) 2 wt % PNIPAAm (PN2-TR); and (**c**) 4 wt % PNIPAAm (PN4-TR).

The distribution of enzymes on the surface of PVDF/nylon-6,6/PNIPAAm and PVDF/nylon-6,6 membranes were analysed using the SEM imaging and shown in Figure 2. All the TR immobilized membranes with no PNIPAAm, 2 and 4 wt % PNIPAAm showed homogenous nanofiber structure with an average nanofiber diameter of 87 ± 17 nm, 180 ± 15 nm and 314 ± 20 nm, respectively. The membrane with 4 wt % PNIPAAm show nano-branched structure with beads and clusters in some nanofibers that could be attributed to the uneven distribution of enzymes; while the membranes with no PNIPAAm and 2 wt % PNIPAAm showed homogenous enzyme attachment as seen in Figure 2. These clusters were formed due to possible aggregation of TR by randomized attachment points on the membrane implying the lack of control on enzyme immobilization [37]. Further, the thickness of the biocatalytic membranes with no PNIPAAm, 2 and 4 wt % PNIPAAm was measured from the cross sectional SEM micrographs to be 249 ± 9 µm, 257 ± 6 µm and 265 ± 11 µm, respectively.

### 3.2. Surface Density of Immobilized Enzyme

**Figure 3:**
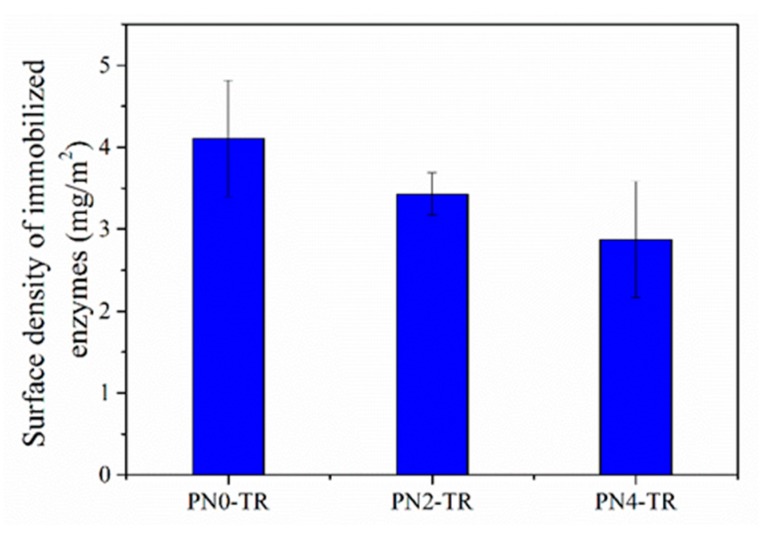
Surface densities of TR immobilized on to PVDF/nylon-6,6/PNIPAAm membranes with no PNIPAAm (PN0-TR), 2 wt % (PN2-TR) and 4 wt % (PN4-TR) PNIPAAm concentrations.

The density of immobilized TR on the surface of membranes was measured to study the amount of covalently attached enzymes and the results are presented in Figure 3. It was observed that the surface density of immobilized TR decreased as the PNIPAAm concentration in the membrane increased. This can be attributed to the incorporation of PNIPAAm in to the membrane which decreased the availability of surface amine functional groups from nylon-6,6 used for enzyme attachment via carbodiimide chemistry using EDC and NHS. The surface densities of immobilized TR on PVDF/nylon-6,6/PNIPAAm membranes with no PNIPAAm, 2 and 4 wt % PNIPAAm were 4.01 mg/m^2^, 3.43 mg/m^2^ and 2.87 mg/m^2^, respectively, which were higher than the reported values of 0.7 mg/m^2^ of TR immobilized PES membrane in the literature due to the nanofiber structure providing a higher surface area for enhanced immobilization [1]. Among the prepared membranes, the control membrane without PNIPAAm had higher surface density of enzymes.

### 3.3. Membrane Characterization

**Figure 4:**
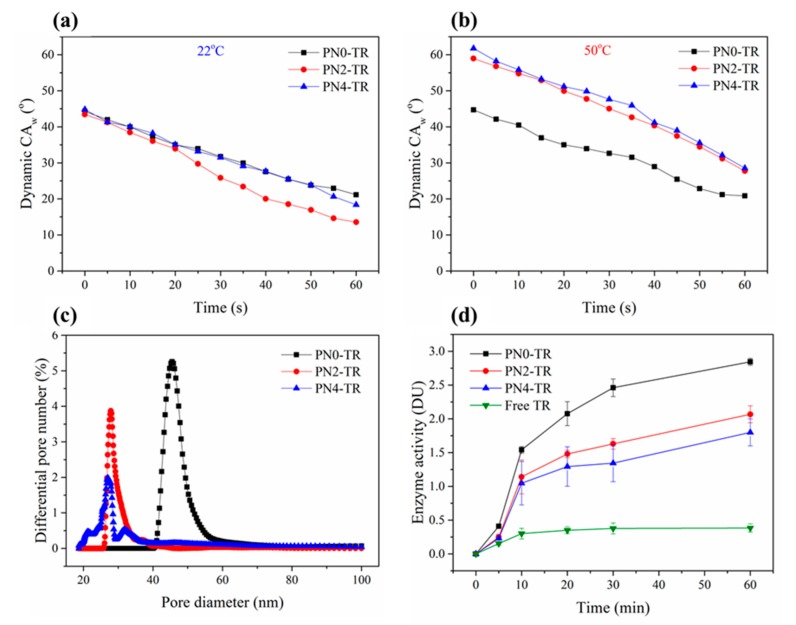
Dynamic water contact angles (CAw) of the biocatalytic membranes with and without PNIPAAm for 60 s contact time at (**a**) 22 °C (Error bars are in the range 0.56−0.89°); (**b**) 50 °C (Error bars are in the range 0.45–1.2°); (**c**) differential pore number (in %) distributions (Error bars are in the range 0.2–0.6%); (**d**) enzymatic activities of biocatalytic membranes over time with no PNIPAAm, 2 and 4 wt % PNIPAAm.

To evaluate the hydrophilicity and responsivity of biocatalytic thermo-responsive membranes, the dynamic water contact angles (CA_w_) were measured over 60 s at 22 °C and 50 °C and are given in Figure 4a,b, respectively. The CA_w_ for the PNIPAAm containing membranes at 22 °C exhibit a slightly faster attenuation compared to control membrane, as shown in Figure 4a. This decreasing tendency could be due to the addition of PNIPAAm that has a hydrophilic extended conformation below its LCST (32 °C) which absorbs water by forming hydrogen bonds between the amide groups of PNIPAAm and water, in spite of having lesser immobilized enzymes compared to control membrane. Also, at 22 °C, the biocatalytic PVDF/nylon-6,6/PNIPAAm membrane with 2 wt % PNIPAAm showed the lowest CA_w_ of 13.6° compared to the membrane with 4 wt % PNIPAAm (18.4°) after 60 s, which may be ascribed to the increased amount of immobilized TR on the membrane surface. Figure 4b shows the dynamic CA_w_ of the as-prepared membranes at 50 °C. For the PVDF/nylon-6,6 without PNIPAAm, the CA_w_ attenuation was similar at both 22 °C and 50 °C. However, the initial CA_w_ values for PNIPAAm containing membranes were higher at 50 °C compared to those at 22 °C, owing to the hydrophobic nature of the membrane above LCST that breaks the hydrogen bonds between amide groups of PNIPAAm and water molecules. 

To investigate the volume-phase transition of the PNIPAAm around its LCST, the thermo-switchable CA_w_ of the membranes was measured and compared in terms of initial CA_w_ at 22 °C and 50 °C, as shown in Figure 4a,b, respectively. The biocatalytic membrane without PNIPAAm exhibited no CA_w_ switchability; while the membranes with 2 and 4 wt % PNIPAAm exhibited switchable CA_w_ from 43.5° to 59° and from 44.8° to 61.8°, respectively, between 22 °C and 50 °C. The slightly higher switchability of biocatalytic membrane with 4 wt % PNIPAAm compared to membrane with 2 wt % PNIPAAm is attributed to increased PNIPAAm concentration in the membrane. However, this CA_w_ variation is more significant than the PVDF-g-PNIPAAm membrane reported in literature that exhibited switching CA_w_ from 87.5° (22 °C ) to 89° (50 °C) [38].

The mean pore size and the distribution of the as-prepared composite membranes were measured by a capillary-flow porometer [5]. The differential pore distributions of the membranes are presented and compared in terms of pore diameters in Figure 4c. The TR immobilized PVDF/nylon-6,6 membrane exhibited narrow distribution curve due to the homogenously attached enzymes; while the TR immobilized membranes with 2 and 4 wt % PNIPAAm exhibited bimodal distribution curves owing to the formation of non-homogenous pore structures due to TR immobilization. The TR immobilized membrane with 4 wt % PNIPAAm membrane showed slightly wider distribution, possibly due to the clustering of TR enzymes as observed in Figure 2. The mean pore size of the TR immobilized on PVDF/nylon-6,6/PNIPAAm membranes with no PNIPAAm, 2 and 4 wt % PNIPAAm were 44, 33 and 23 nm, respectively. The smaller pore size of the as-prepared membrane with 4 wt % PNIPAAm compared to those membranes with no PNIPAAm and 2 wt % PNIPAAm is ascribed to the formation of enzyme clusters on the membrane surface (Figure 2c). 

### 3.4. Enzyme Activity Evaluation Across the Nano-Composite Membranes

Figure 4d show the results respective to the reaction time. The number of products formed by immobilized TR were noticed to be much greater than that of the free enzymes for all reaction times up to 60 min. For instance, at 60 min, the TR immobilized on to the membranes with no PNIPAAm, 2 and 4 wt % PNIPAAm produced about 7.5, 5.5 and 4.7 times more peptide products, respectively, than the free TR. It was also observed that the activity of immobilized TR increased with reaction time; while the activity of free enzymes increased initially but became stable in 10 min. This is due to the increased stability of immobilized TR that has greatly enhanced the enzymatic activity, whereas the free TR undergoes autolytic behaviour commonly known as self-digestion [39,40,41]. The results further revealed that the PVDF/nylon-6,6 membrane without PNIPAAm show superior enzyme activity than the PNIPAAm containing membranes, possibly due to high immobilization density (Figure 3). 

### 3.5. Protein Fouling Studies

The combined enzymatic and thermo-responsive effect on surface–protein interaction of the as-prepared biocatalytic membranes was investigated by conducting the filtration experiments with and without temperature-change cleaning, i.e., two-cycle filtration with respective intermediate DI water cleaning at 22 °C and 50 °C.

**Figure 5:**
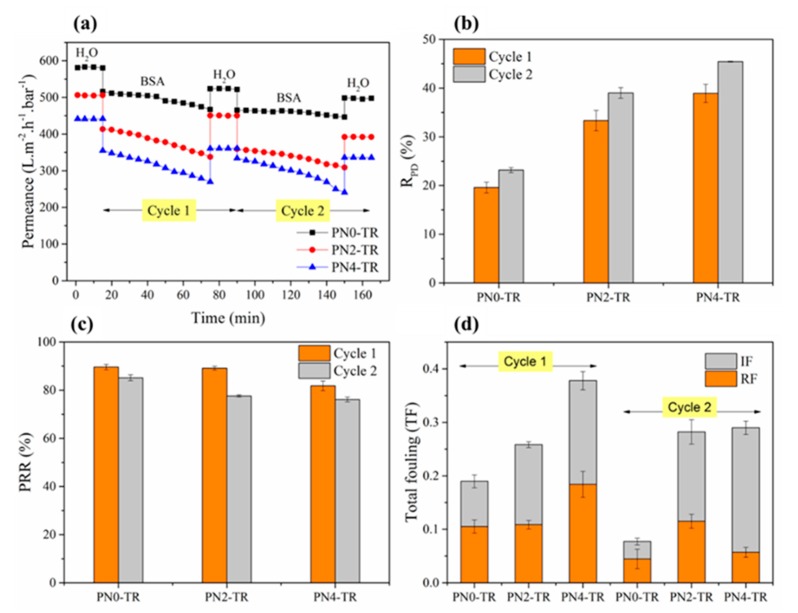
Protein fouling studies for biocatalytic membranes with and without PNIPAAm. (**a**) Permeance values for two filtration cycles; (**b**) R_PD_ after each filtration cycle; (**c**) PRR after each filtration cycle; (**d**) RF, IF and TF for 2 filtration cycles. Experimental Conditions: Pressure = 100 kPa, cross-flow velocity = 12.6 cm/s, feed solution = 1 g/L BSA, 1 mM CaCl_2_, 7 mM NaCl, both filtration and cleaning temperature = 22 °C.

Figure 5 shows the results of two consecutive filtration cycles with intermediate DI water cleaning at 22 °C presented in terms of water permeance and R_PD_ as a measure of protein fouling, and PRR, IF, RF and TF, as measures of the self-cleaning ability of the membranes. The error bars in Figure 5 indicate the reproducibility of the results. As presented in Figure 5a, the biocatalytic membranes with 2 wt % (506 L m^−2^ h^−1^ bar^−1^) and 4 wt % (442 L m^−2^ h^−1^ bar^−1^) PNIPAAm exhibited slightly lower initial water permeance i.e., 13% and 24% lesser, compared to the membrane without PNIPAAm (581 L m^−2^ h^−1^ bar^−1^), which is attributed to the decrease in pore size due to the incorporation of PNIPAAm (Figure 4). Based on the permeance patterns observed for all membranes in Figure 5a, the R_PD_ was calculated based on Equation (2) and presented in Figure 5b to indicate the resistance to protein fouling. During the first filtration cycle, the biocatalytic PVDF/nylon-6,6/PNIPAAm membranes with no PNIPAAm, 2 and 4 wt % PNIPAAm suffered fouling as indicated by an R_PD_ of about 19%, 33% and 39%, respectively. The lower R_PD_ of biocatalytic membrane without PNIPAAm suggests that the membrane with higher density of immobilized enzymes with increased proteolytic ability i.e., protein digestive feature, were able resist BSA fouling to a larger extent [39]. Also, this result was found to be promising compared to the TR immobilized PMAA-g-PES UF membrane as reported in literature that showed 19.1% flux decline rate using 1 g/L BSA solution [1]. 

**Figure 6:**
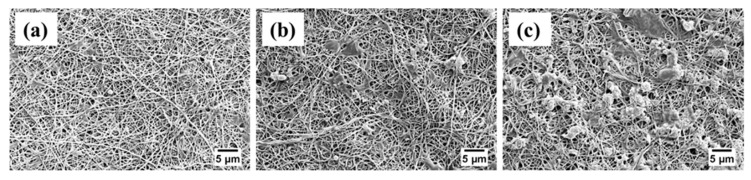
SEM micrographs of BSA fouled biocatalytic membranes with (**a**) no PNIPAAm (PN0-TR); (**b**) 2 wt % (PN2-TR); and (**c**) 4 wt % (PN4-TR) PNIPAAm after two filtration and cleaning cycles at 22 °C.

Further, during the second filtration cycle, the R_PD_ values were 22%, 39% and 45% for respective biocatalytic membranes with no PNIPAAm, 2 and 4 wt % PNIPAAm, after temperature cleaning at 22 °C. Similar to first filtration cycle, the increasing R_PD_ follows the decreasing trend of immobilized TR density on the membrane surface. The SEM micrographs of the fouled membranes are presented in Figure 6. Consistent to the permeance results, the biocatalytic PVDF/nylon-6,6/PNIPAAm membrane with 4 wt % PNIPAAm showed heavy fouling (Figure 6c) compared to that without PNIPAAm that exhibited much reduced protein deposition presenting clear surface after two filtration cycles (Figure 6a), followed by the membrane with 2 wt % PNIPAAm that showed regional accumulation of protein (Figure 6b).

The self-cleaning efficiency of the as-prepared biocatalytic membranes without temperature cleaning was quantified by computing PRR and fouling parameters namely IF, RF and TF. Figure 5c reveals that after the first filtration cycle, the biocatalytic membranes with no PNIPAAm, 2 and 4 wt % PNIPAAm were able to recover about 90%, 89% and 82% of the initial permeance, respectively. The greater permeance recovery of membranes with no PNIPAAm and 2 wt % PNIPAAm compared to that with 4 wt % PNIPAAm was attributed to the higher density of immobilized enzymes on the membrane surface that leads to breakdown of proteins into smaller polypeptides releasing them subsequently from the membrane surface. This result was found to be comparable with the TR immobilized PVDF MF membrane constructed using a complex method involving electron beam that showed 90% flux recovery after first filtration cycle with pure BSA solution of 3 g/L after backwashing with 120 mL of pure water every 1.6 L of filtration and self-cleaning through trypsin activation by immersing the fouled membrane into a buffered solution at 37 °C and pH 8.0 overnight [27]. Similar trend was observed after the second filtration cycle with biocatalytic membranes with no PNIPAAm, 2 and 4 wt % PNIPAAm showing 85%, 78% and 76% permeance recovery, respectively. The corresponding IF and RF parameters are presented in Figure 5d. After the first filtration cycle, the membranes with no PNIPAAm and 2 wt % PNIPAAm reduced the IF by 43% and 41%, respectively, compared to that with 4 wt % PNIPAAm, explaining the higher PRR presented in Figure 5c. This result demonstrates that less permanent fouling occurs with more enzymes featuring the self-cleaning capacity of the biocatalytic membranes. Thus, the membranes with higher density of immobilized enzymes exhibited much lower TF, which is corresponding to their higher PRR. Here, depending on the self-cleaning ability and fouling mitigation, the biocatalytic PVDF/nylon-6,6 membrane without PNIPAAm was recognised as the best performing membrane. 

**Figure 7:**
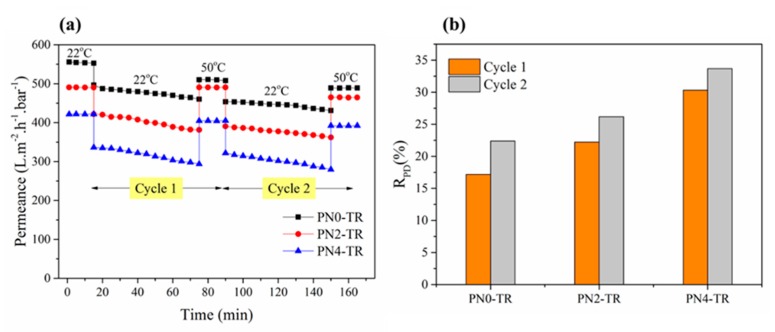
Protein fouling studies for biocatalytic membranes with and without PNIPAAm. (**a**) Permeance values for two filtration cycles; (**b**) R_PD_ after each filtration cycle (Error bars are in the range 1.1–2.9°). Experimental Conditions: Pressure = 100 kPa, cross-flow velocity = 12.6 cm/s, feed solution = 1 g/L BSA, 1 mM CaCl_2_, 7 mM NaCl, filtration temperature = 22 °C, cleaning temperature = 50 °C.

To investigate the effect of PNIPAAm in the membrane matrix, the as-prepared biocatalytic PNIPAAm membranes were evaluated with the same filtration experiments, but involved temperature-change cleaning with DI water at 50 °C. The performance results in terms of permeance and R_PD_ for two filtration cycles are given in Figure 7a,b, respectively. As shown in Figure 7a, the biocatalytic membranes with no PNIPAAm (556 L m^−2^ h^−1^ bar^−1^), 2 wt % (491 L m^−2^ h^−1^ bar^−1^) and 4 wt % (422 L m^−2^ h^−1^ bar^−1^) exhibited similar initial water permeance to those presented in Figure 5a, showing good repeatability. Also, these values were found to be higher than the initial water permeance (74.3 L m^−2^ h^−1^ bar^−1^) of PNIPAAm-g-ZrO_2_ membrane reported in literature [34]. During the first filtration cycle, the R_PD_ values for biocatalytic PVDF/nylon-6,6/PNIPAAm membranes with no PNIPAAm, 2 and 4 wt % PNIPAAm were 18%, 22% and 30%, which are found to be greater than the 10.9% reduction of flux of PNIPAAm-g-ZrO_2_ membrane reported in literature [34]. Further, during the second filtration cycle, the R_PD_ values were 22%, 26% and 33% for the respective membranes. The increasing trends of the R_PD_ in both cycles are consistent with those in Figure 3 corresponding to increasing density of enzymes on the membrane surface. Nevertheless, these values were found to be lower than the R_PD_ values reported with intermediate cleaning at 22 °C in Figure 5b. Also, from Figure 7a, during the second filtration cycle, the membranes with no PNIPAAm, 2 wt % and 4 wt % PNIPAAm recovered about 91%, 93% and 96% of the initial BSA permeance of first filtration cycle. Thus, in addition to the enzymatic protein digestive feature of the membrane, the temperature-change cleaning has confirmed the role of PNIPAAm on the antifouling and self-cleaning effects via thermo-switchable cleaning when the environment temperature switches from 22 °C to 50 °C. Overall, the as-prepared biocatalytic membrane without PNIPAAm revealed superior fouling resistance with reduced protein interactions compared to PNIPAAm containing membranes, indicating that higher degree of enzyme immobilization offers better self-cleaning than the combined effect at low enzyme and PNIPAAm concentrations. However, enzymes may suffer from deteriorating performance due to loss in biocatalytic activity over time [9,28] and hence further optimization of PNIPAAm concentration could be performed to achieve maximum thermo-switchable feature that further enhances the self-cleaning efficiency of membranes.

### 3.6. Storage Studies & Effect of Thermo-Responsivity on Enzyme Stability

**Figure 8:**
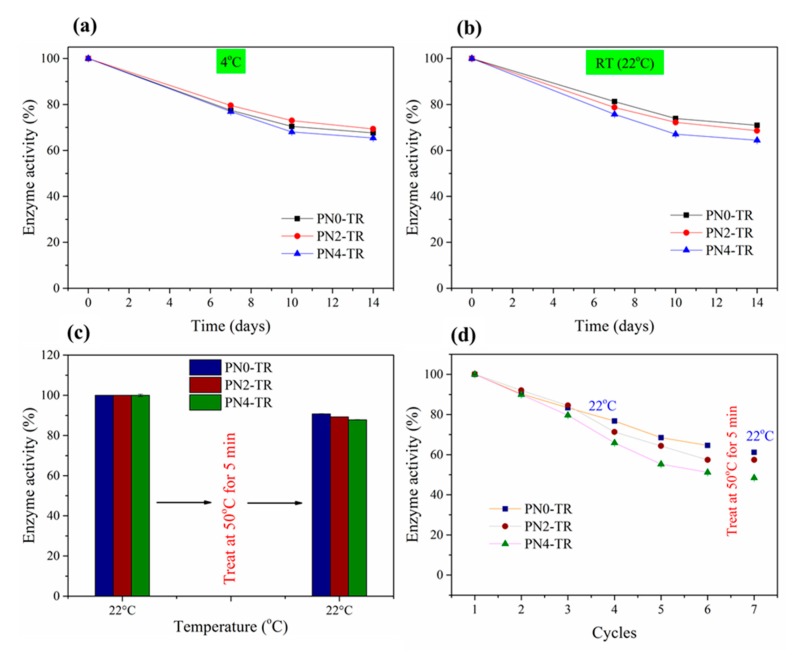
Hydrolytic activities of biocatalytic membranes for up to 14 days of storage at (**a**) 4 °C and (**b**) 22 °C; Stability of enzymes immobilized on to membranes in terms of enzyme activity with 50 °C treatment for 5 min after (**c**) one reuse cycle and (**d**) six reuse cycles.

The effect of storage time on the hydrolytic activities of the immobilized TR at 4 °C and RT (22 °C) were presented in Figure 8a,b, respectively. It was revealed that at both RT and 4 °C, the biocatalytic membrane without PNIPAAm retained about 81% and 78% of their initial enzymatic activities after 7 days, respectively, and about 71% and 69% of their initial activities after 14 days of storage. The activity results were found to be similar to the TR immobilized PVDF/nylon-6,6/chitosan membrane that was prepared in our earlier study [9] with 81% (RT) and 70% (4 °C) detainment of initial enzyme activity after 7 and 14 days of storage, respectively, showing good reproducibility. Thus, the prepared membranes may not require inconvenient refrigerated storage conditions and can be stored at RT. Similarly, the membranes with 2 and 4 wt % PNIPAAm stored at RT retained about 79% and 76% of the activity after 7 days, respectively, and about 69% and 64% of the initial activity after 14 days, respectively. 

The effect of thermo-switchable volume phase transition of the as-prepared membranes on the activities of freshly immobilized and used TR enzymes was investigated and the respective results are given in Figure 8c,d. In Figure 8c, the enzyme activities of biocatalytic membranes with no PNIPAAm, 2 and 4 wt % PNIPAAm declined only about 9%, 11% and 12% after treating at 50 °C, which is similar to the storage data (Figure 8a,b) that did not affect the immobilized enzymes of PNIPAAm membranes. The enzyme activity of membrane with 4 wt % PNIPAAm declined most significantly by 12%, which is more than that without PNIPAAm (9%), possibly owing to the leaching of weakly attached TR enzyme clusters formed through aggregation on the membrane surface as observed in Figure 2. Similarly, in Figure 8d, the enzyme activities of as-prepared membranes after six consecutive reuse cycles and treatment at 50 °C dropped less than about 3% after treating at 50 °C. This could be due to the stable enzyme activity at both 22 °C and 50 °C temperatures and during conformational volume phase transition when the temperature switches from 22 °C to 50 °C. Further, from Figure 8d, the hydrolytic activities of immobilized enzymes declined with increasing reuse cycles (up to six cycles), that may be due to (a) the release of any enzymes that are weakly bound and (b) the gradual morphological change of fibers including swelling and disintegration due to high hydrophilicity [28]. Also, the biocatalytic membranes with 2 and 4 wt % PNIPAAm show faster decline compared to the PNIPAAm-free membrane which may also be due to the loss of enzyme activity via change in nanofiber morphology via swelling and disintegration. Thus, the thermo-switchable volume phase transition of the as-prepared membranes was not found to affect the enzyme activity that was stable when temperature switched from 22 °C to 50 °C. 

## 4. Conclusions

Biocatalytic membranes with and without PNIPAAm were successfully prepared by immobilizing trypsin enzymes onto a hydrophilic nylon-6,6/PNIPAAm nanofiber layer supported by a hydrophobic PVDF cast layer. It was demonstrated that superior enzyme loading on to the membrane without PNIPAAm can be achieved compared to PNIPAAm-containing membranes, owing to the amine-rich nanofibrous surface with high surface-to-volume ratio. The trypsin-immobilized membranes minimized surface–protein contamination on the surface via enzyme proteolytic digestion. As a result of the UF study conducted using model feed solution containing BSA, CaCl_2_ and NaCl, the biocatalytic membrane without PNIPAAm offered superior performance in separation and purification applications with more permeability and less fouling than the other membranes with PNIPAAm, demonstrating that higher degree of enzyme immobilization offers better self-cleaning than the combined self-cleaning of low concentrations of enzyme and PNIPAAm. Also, the thermo-switchable conformational volume phase transition of the as-prepared membranes did not affect the stability of surface immobilized enzymes. Hence, the fabrication of biocatalytic nanofibrous surface has greater potential to mitigate fouling and self-clean the fouled surfaces beyond membrane separation. 

## Abbreviations

PVDF	poly(vinylidene fluoride)
PNIPAAm	poly(*N*-isopropylacrylamide)
BSA	bovine serum albumin
NaCl	sodium chloride
CaCl_2_	calcium chloride
UF	ultrafiltration
PET	polyethylene terephthalate
PLA	poly(lactic acid)
LCST	lower critical solution temperature
TiO_2_	titanium dioxide
ZrO_2_	zirconium dioxide
TR	trypsin
PVP	poly(vinyl pyrrolidone)
EDC	1-ethyl-3-(3-dimethylaminopropyl) carbodiimide
NHS	*N*-hydroxysuccinimide
DMAC	*N*,*N*′-dimethylacetamide
TCA	trichloroacetic acid
DI	deionised
SEM	scanning electron microscopy
CA_w_	water contact angle
DU	digestion unit
R_PD_	rate of permeance decline
PRR	permeance recovery rate
